# *In vivo* Cross-Linking MS of the Complement System MAC Assembled on Live Gram-Positive Bacteria

**DOI:** 10.3389/fgene.2020.612475

**Published:** 2021-01-08

**Authors:** Hamed Khakzad, Lotta Happonen, Guy Tran Van Nhieu, Johan Malmström, Lars Malmström

**Affiliations:** ^1^Equipe Signalisation Calcique et Infections Microbiennes, Ecole Normale Supérieure Paris-Saclay, Gif-sur-Yvette, France; ^2^Institut National de la Santé et de la Recherche Médicale U1282, Gif-sur-Yvette, France; ^3^Faculty of Medicine, Department of Clinical Sciences, Division of Infection Medicine, Lund University, Lund, Sweden

**Keywords:** membrane attack complex, cross-linking mass spectrometry, *Streptococcus pyogenes*, Gram-positive bacteria, *in vivo* cross-linking

## Abstract

Protein–protein interactions are central in many biological processes, but they are challenging to characterize, especially in complex samples. Protein cross-linking combined with mass spectrometry (MS) and computational modeling is gaining increased recognition as a viable tool in protein interaction studies. Here, we provide insights into the structure of the multicomponent human complement system membrane attack complex (MAC) using *in vivo* cross-linking MS combined with computational macromolecular modeling. We developed an affinity procedure followed by chemical cross-linking on human blood plasma using live *Streptococcus pyogenes* to enrich for native MAC associated with the bacterial surface. In this highly complex sample, we identified over 100 cross-linked lysine–lysine pairs between different MAC components that enabled us to present a quaternary model of the assembled MAC in its native environment. Demonstrating the validity of our approach, this MAC model is supported by existing X-ray crystallographic and electron cryo-microscopic models. This approach allows the study of protein–protein interactions in native environment mimicking their natural milieu. Its high potential in assisting and refining data interpretation in electron cryo-tomographic experiments will be discussed.

## Introduction

The human immune system plays a crucial role in detecting and clearing bacterial infections. The complement system is an important immunological sensor and effector pathway that in interplay with other components of innate and adaptive immunity defends the host against infection and regulates the inflammatory response. Complement activation can occur through three distinct pathways depending on the molecular trigger, all proceeding through a proteolytic cascade that leads to the formation of the multicomponent membrane attack complex (MAC) capable of disrupting target cell membranes. While Gram-positive bacteria are thought to be able to resist the MAC-induced membrane disruption due to their thick cell wall, several such bacteria have also been shown to secrete small proteins that specifically target this multicomponent complex (Berends et al., 2013) and to possibly inhibit its formation and function. In *Streptococcus pyogenes*, *SIC* (Akesson et al., 1996) and the recently characterized ISP (Happonen et al., 2019) perform this function.

The importance of MAC targeting the surface of Gram-positive bacteria is illustrated in various examples. Berends et al. (2013) found that complement activation leads to specific C3-independent deposition of MAC on the Gram-positive bacterial surface. In *S. pyogenes*, this deposition is localized close to the division septum (Berends et al., 2013). Intriguingly, the mammalian peptidoglycan recognition proteins (PGRPs) bind to the bacterial cell wall of Gram-positive *Bacillus subtilis* and kill the bacteria through specific interaction with the CssR–CssS two-component system at the site of daughter cell separation (Kashyap et al., 2011). While we previously reported interactions between MAC components and bacterial CovR, part of CovRS two-component system in *S. pyogenes* (Happonen et al., 2019), the importance of studying the MAC formation on the bacterial surface is evident.

**FIGURE 1:**
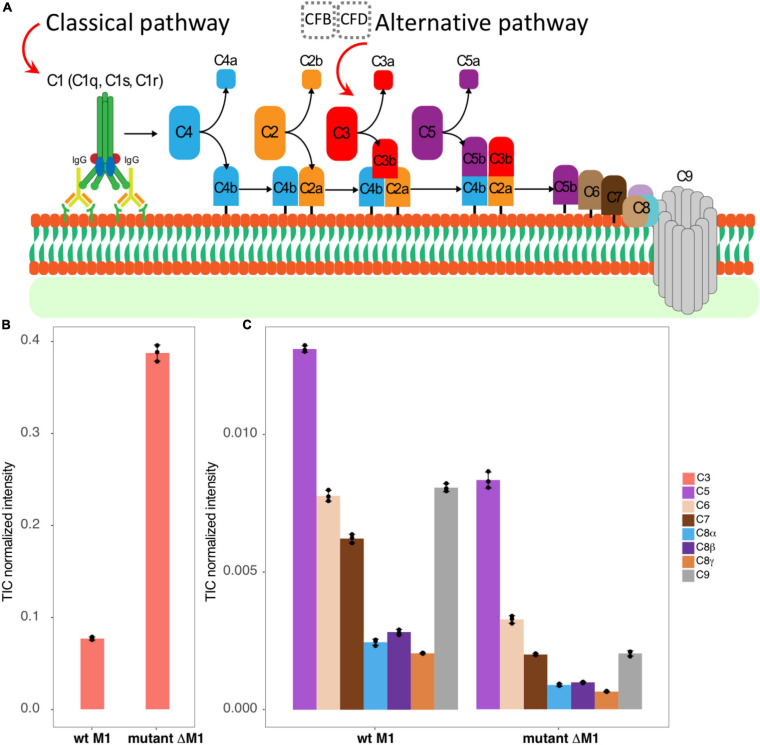
The studied system. **(A)** A schematic view represents briefly how classical and alternative complement pathways activated by antigen–antibody interaction, or by factors B and D, lead to C3 convertase production that eventually brings the MAC C5b–C9 components to assemble as a pore on the surface of the bacteria. **(B,C)** The bar graphs represent TIC-normalized intensity of complement proteins in human plasma adsorption samples. It provides a comparison between the wt strain SF370 (left) and the mutant strain ΔM1 (right) for C3 **(B)**, C5, C6, C7, C8, and C9 components **(C)**.

The MAC consists of four single-copy components, C5b, C6, C7, and C8, together with multiple copies of C9, the structural component forming the pore walls (Figure 1A). The MAC has been extensively studied, and the structure has been determined at resolutions ranging from 2.51 to 5.6 Å. Many structures of individual components, partial assemblies, and full assemblies are available (Lovelace et al., 2011; Serna et al., 2016; Menny et al., 2018).

Biological mass spectrometry (MS) has undergone rapid evolution and has become the method of choice to analyze complex protein samples (Aebersold and Mann, 2003). One of the most powerful features of MS is handling complex samples with ease, permitting the identification and quantification of proteins in biopsies and other clinical samples (Guo et al., 2015; Zhang et al., 2019). Other important aspects are its high capacity, a high degree of automated operation, and reliability. Modern instruments and acquisition strategies can identify and quantify thousands of proteins with high accuracy and high sensitivity (Röst et al., 2014). The data obtained by MS can be categorized depending on acquisition strategies into high-resolution MS1 proteomics (hrMS1), shotgun proteomics, or data-dependent acquisition (DDA) and data-independent acquisition (DIA). Shotgun proteomics measure the m/z of all peptide ions introduced into the MS, the produced spectra being called an MS1 or precursor spectra, then select up to tens of prominent ions, which are isolated and fragmented. The m/z of all produced fragments is measured (the produced spectra referred to as MS2 or tandem spectra) (Nesvizhskii and Aebersold, 2005). In hrMS1, the MS only measures MS1 spectra at the highest possible resolution. In DIA acquisition, systematic fragment intensities are collected with many peptides being fragmented and measured at once. This results in throughput to levels similar to shotgun MS but increases the quantitative accuracy (Gillet et al., 2012).

Cross-linking MS (XL-MS) has been shown to be a powerful technique to measure protein–protein interactions directly in complex samples (Sinz, 2006; Herzog et al., 2012; Liu et al., 2015; Hauri et al., 2019). Here, a bi-functional cross-linking reagent, such as disuccinimidyl suberate (DSS), is used to covalently link two lysine residues when the proteins are in their native states. The proteins are subjected to endoproteolytic digestion using, for example, trypsin, and peptides linked by DSS are analyzed. Cross-links between polypeptide chains are called inter-cross-links, as opposed to intra-cross-links. As the proteins are in their native state when cross-linked, the 11.4 Å long DSS arm determines the maximum distance between the cross-linked amino acids. The maximum distance information is then used to identify and characterize protein–protein interactions. A model of the protein–protein interaction can be created using macromolecular docking software, such as RosettaDock (Gray, 2006; Koehler Leman et al., 2020), MEGADOCK (Ohue et al., 2014b), or HADDOCK (Zundert et al., 2016), in cases where enough cross-links are identified and reliable models of the interacting proteins are available.

Here, we applied a simplified version (see the Materials and Methods section for details) of the recently developed targeted cross-linking mass spectrometry (TX-MS) approach (Hauri et al., 2019) to study the formation of MAC on the surface of live *S. pyogenes* bacteria in human plasma. We show that our integrative mass spectrometric approach can detect cross-linked peptides in complex unfractionated samples. This integrative approach is based on MS/MS acquisitions filtered by hrMS1 isotopic patterns combined with macromolecular docking encompassing the entire MAC and recapitulating the interaction interfaces previously described by X-ray crystallography (Lovelace et al., 2011) and single-particle electron cryo-microscopy (Serna et al., 2016; Menny et al., 2018). These results further highlight that MAC can assemble in its active, pore-like form on the surface of Gram-positive bacteria with identified cross-linked peptides between adjacent C9 proteins.

## Materials and Methods

### Computational Workflow

The UniProt accession IDs for human complement proteins considered in this study, including C3, C5, C6, C7, C8α, C8β, C8γ, and C9, were P01024, P01031, P13671, P10643, P07357, P07358, P07360, and P02748, respectively. The computational modeling method designed for this study was a modified version of the TX-MS workflow (Hauri et al., 2019). Accordingly, we first generated a list of all hypothetical lysine–lysine pairs based on the sequence of partner proteins for each pairwise protein–protein interaction. Considering all studied components and with the minimum peptide length restricted to five residues, this list included a total number of 27,873 hypothetical XLs.

The generated XL list was analyzed for each interface through all MS/MS, DDA samples using the TX-MS workflow. To do that, DDA mzML data were first converted to Mascot Generic Format (MGF) files using msconvert (Chambers, 2012). Then, precursor m/z values for each XL were calculated in six different charge states ranging from + 3 to + 8. These values were used to filter the MGF files with a delta window of 0.05 m/z. After that, a list of product ions (y and b ions with charge states + 1 to + 3) were produced for each selected XL and searched through all filtered spectra to find a similar pattern. Finally, by considering the number of detected fragment ions, those spectra with >30 matched product ions were selected for the second phase and to be validated by hrMS1 data.

To further validate selected XLs from DDA samples, we used Dinosaur software (Teleman et al., 2016) to extract the isotopic pattern for each selected XL and searched them through hrMS1 samples. The final filtered list of XLs (those detected in hrMS1 data) in this stage was used as distance constraints for protein–protein docking. For structural docking, we used the input protein structures deposited in protein data bank with PDB IDs: 6H04, 2WII, 3OJY, and 4A5W.

To generate docking models, we used MEGADOCK 4.0, a fast Fourier transform (FFT)-based rigid-body docking software (Ohue et al., 2014b). Its scoring function is a combinatorial score based on electrostatics and desolvation free energy combined with shape complementarity (Ohue et al., 2014a). We produced 2,000 docking models per interface. So, by considering the six evaluated pairwise interfaces, a total number of 12,000 docking models were generated where for each interface, the top 100 models based on the MEGADOCK score were selected and then filtered out using distance restraints provided by XLs. The threshold cut-off to evaluate models based on XLs was set to 32 Å between Cβ–Cβ as we used DSS (with 11.4 Å arm length) to prepare XL-MS samples. Finally, the top five models based on the number of XLs they fulfill and the average length of all fulfilled XLs in the model were selected as the final models.

### Cross-Linking of Plasma Adsorption Samples

For cross-linking of MAC to the surface of *S. pyogenes*, we used pooled normal human plasma adsorbed onto the surface of *S. pyogenes* bacteria (Happonen et al., 2019; Hauri et al., 2019). Briefly, the *S. pyogenes* M1 serotype strain SF370 from the American Type Culture Collection (ATCC; strain reference 700294) and its derived ΔM1 mutant lacking the major surface antigen, the M1 protein, was grown at 37°C 5% CO_2_ to mid-exponential phase (OD_620 nm_ ∼0.4–0.5) in Todd–Hewitt (TH) broth supplemented with 0.3% (w/v) yeast extract. The cells were harvested by centrifugation (1,900 × *g*, 10 min, 22°C), washed once with phosphate-buffered saline [PBS; 10 mM phosphate buffer, 2.7 mM potassium chloride, 137 mM sodium chloride (Sigma)], recentrifuged (1,900 × *g*, 5 min, 22°C), and resuspended to an approximate concentration of 1 × 10^9^ colony forming units ml^–1^. For plasma adsorption, 400 μl of pooled normal human plasma from healthy donors (Innovative Research) was supplemented with a final concentration of 10 μM argatroban (Sigma) to prevent any plasma clotting and was mixed with 100 μl of bacteria with subsequent incubation at 37°C for 30 min at 500 rpm.

SF370 and ΔM1 bacteria with adsorbed plasma proteins–including the MAC–were harvested by centrifugation (1,900 × *g*, 5 min, 22°C), washed twice with PBS, and finally resuspended in 200 μl of PBS for cross-linking. For cross-linking, heavy/light disuccinimidyl-suberate cross-linker (DSS-H12/D12, Creative Molecules Inc.) resuspended in 100% dimethylformamide (Sigma) was added to final concentrations of 0, 0.25, 0.5, 1.0, and 2.0 mM in duplicates, and the samples were incubated at 37°C for 1 h at 900 rpm. The cross-linking reaction was quenched with a final concentration of 50 mM ammonium bicarbonate (Sigma) at 37°C for 15 min at 900 rpm. The MAC complex adsorbed to the bacterial surface was released by limited proteolysis with 2 μG trypsin (Promega) at 37°C for 1 h at 800 rpm prior to cell debris removal by centrifugation (1,900 × *g*, 15 min) and subsequent supernatant recovery. Two hundred microliters of the supernatant containing the MAC was recovered, and any remaining bacteria were killed by heat inactivation (85°C, 5 min) prior to sample preparation for MS.

### Sample Preparation

For MS, 100 μl of the heat-inactivated supernatant containing the MAC was denatured in 200 μl 8 M urea–100 mM ammonium bicarbonate (both Sigma), and the cysteine bonds were reduced with 5 mM *tris*(2-carboxyethyl)phosphine (Sigma) at 37°C for 2 h at 500 rpm. The cysteines were alkylated with 5 mM iodoacetamide (Sigma) at 22°C for 30 min, and the samples were subsequently digested using sequencing-grade lysyl endopeptidase (Wako) at 37°C for 4 h at 500 rpm. The samples were diluted with 100 mM ammonium bicarbonate to a final urea concentration of 1.5 M and subsequent trypsin (Promega) digestion at 37°C for 16 h. Digested samples were acidified with 10% formic acid to a final pH of 3.0, and the peptides were purified with C18 reverse-phase spin columns according to the manufacturer’s instructions (Macrospin columns; Harvard Apparatus). Dried peptides were reconstituted in 2% acetonitrile and 0.2% formic acid prior to MS analyses.

### MS for Cross-Linking Analysis

All peptide analyses were performed on a Q Exactive HFX mass spectrometer (Thermo Scientific) connected to an EASY-nLC 1200 ultra-high-performance liquid chromatography system (Thermo Scientific). For analysis of cross-linked samples, we used DDA-MS and hrMS1. For DDA analysis, the peptides were separated on an EASY-Spray column (Thermo Scientific; ID 75 μm × 25 cm, column temperature 45°C) operated at a constant pressure of 800 bar. A linear gradient from 4 to 45% of 0.1% formic acid in 80% acetonitrile was run for 50 min at a flow rate of 300 nl min^–1^. One full MS scan (resolution 60,000 at 200 m/z; mass range 350–1,600 m/z) was followed by MS/MS scans (resolution 15,000 at 200 m/z) of the 15 most abundant ion signals. The precursor ions were isolated with 2 m/z isolation width and fragmented using higher-energy collisional-induced dissociation (CID) at a normalized collision energy (NCE) of 30. Charge state screening was enabled, and precursors with an unknown charge state and singly charged ions were excluded. The dynamic exclusion window was set to 15 s and limited to 300 entries. The automatic gain control was set to 3e6 for MS and 1e5 for MS/MS with ion accumulation times of 110 and 60 ms, respectively. The intensity threshold for precursor ion selection was set to 1.7e4.

In the hrMS1 analysis, peptides were separated using an EASY-Spray column (Thermo Scientific; ID 75 μm × 25 cm, column temperature 45°C) operated at a constant pressure of 800 bar. A linear gradient from 4 to 45% of 0.1% formic acid in 80% acetonitrile was run for 60 min at a flow rate of 300 nl min^–1^. High-resolution MS scans (resolution 240,000 at 200 m/z; mass range from 400 to 2,000 m/z) were acquired using automatic gain control set to 3e6 and a fill time of 500 ms.

### MS for Protein Quantification

The MAC enriched to the streptococcal surface was quantified using DIA-MS. For DIA-MS, the peptides from the non-cross-linked samples were separated using an EASY-Spray column (Thermo Scientific; ID 75 μm × 25 cm, column temperature 45°C) operated at a constant pressure of 800 bar. A linear gradient from 4 to 45% of 0.1% formic acid in 80% acetonitrile was run for 110 min at a flow rate of 300 nl min^–1^. A full MS scan (resolution 60,000 at 200 m/z; mass range from 390 to 1,210 m/z) was followed by 32 MS/MS full fragmentation scans (resolution 35,000 at 200 m/z) using an isolation window of 26 m/z (including 0.5 m/z overlap between the previous and next window). The precursor ions within each isolation window were fragmented using higher-energy CID at a NCE of 30. The automatic gain control was set to 3e6 for MS and 1e6 for MS/MS with ion accumulation times of 100 and 120 ms, respectively. For protein abundance comparison across the different streptococcal strains, the data were normalized based on measured total ion current (TIC).

## Results

### The Human MAC Assembles on the Surface of Live Gram-Positive Bacteria

We studied the MAC assembly on the surface of live bacteria. The MAC has previously been shown to associate at the division septum of *S. pyogenes*, where the cell wall might not yet be fully mature, in a complement system C3-independent manner, possibly mediated through the interaction with ISP and CovR (Happonen et al., 2019). Here, we demonstrated that the MAC is assembled on the surface of *S. pyogenes*. In order to study the MAC assembly on a Gram-positive bacterium, we performed TX-MS using human plasma on the surface of live wt *S. pyogenes* bacteria expressing the main surface antigen, the M1 protein, and an isogenic mutant lacking M1 expression, without any subsequent enrichment of cross-linked peptides.

By analyzing non-cross-linked control samples by DIA-MS, we found the complement protein C3 and all MAC components (C5b, C6, C7, C8, and C9) to be associated with the bacterial surface (Figure 1B). Intriguingly, the mutant lacking M1 expression bound complement C3 to a higher degree than the wt strain (Figure 1B). These results could be explained by the lack of steric hindrance imposed by the extended, coiled-coil M1 protein in the M1-deficient strain (Happonen et al., 2019). Moreover, the MAC components were more enriched in the wt strain than in the M1 mutant (Figure 1C), consistent with MAC binding to *S. pyogenes* being complement C3-independent.

We validated the structure of some of the intact components from human blood plasma enriched at the bacterial surface by analyzing intra-protein cross-links. For this, we analyzed the samples cross-linked with DSS. Taken together, we identified 43, 32, and 10 intra-protein cross-links for C3, C5, and all three chains (α, β, and γ) of C8. Supplementary Figure 1 represents the detected XLs mapped on the experimentally solved structures (PDB IDs: 6H04, 3OJY, 4A5W, and 2WII).

### Structural Modeling

**FIGURE 2:**
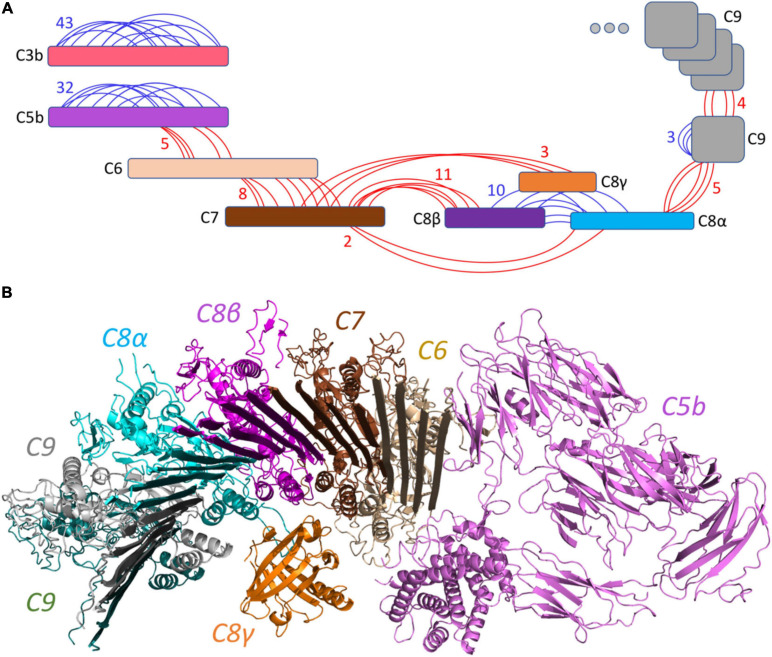
Overview of the identified cross-linked peptides. **(A)** Detected XLs are indicated in red (inter-links) or blue (intra-links). A total number of 126 XLs with an estimated average of 8 links per interface were identified. **(B)** The generated pairwise docking models were filtered out by XL peptides and were structurally aligned together to represent the C5b–C9 complex initiating the pore by multiple C9 components. This model is supported by 38 inter-XLs.

The structure of all MAC components has been determined *in vitro* at different resolutions (Serna et al., 2016; Menny et al., 2018) ranging from 2.51 to 5.6 Å, as indicated in Table 1. To determine the structural model of the complement system MAC in its native environment enriched from human plasma onto live bacteria, we first analyzed the pairwise interactions between the subunits by considering six interfaces including C5b–C6, C6–C7, C7–C8, C8α–C8β, C8 (α, β, and γ)–C9, and C9–C9. For each interface, we generated a list of all hypothetical lysine–lysine pairs (Table 1), which were further investigated using MS data generated by two different mass spectrometric acquisition methods, DDA-MS and hrMS1. Accordingly, by retaining the spectrums with high fragment ion coverages from DDA data, we further evaluated the MS/MS output XL list by hrMS1 data to filter out XLs based on their isotopic pattern (see the Materials and Methods section). Applying this double filtration step, the produced list was used to evaluate molecular docking models of each pairwise interface. As a result, we generated docking models for each interface supported with an estimated average of eight inter-XLs per interface (Figure 2A).

**TABLE 1:** The Identified XLs for individual components of pairwise protein–protein interactions with previously solved structures.

Components	Reference PDB	# K–K PAIRS	# INTRA-XLS	# INTER-XLS
C3B	2WII (X-ray, 2.7 Å)	5,886	43	–
C5B	4A5W (X-ray, 3.5 Å)	6,105	32	–
C5B–C6	6H04 (EM, 5.6 Å)	6,882	–	5
C6–C7	6H04 (EM, 5.6 Å)	2,728	–	8
C7–C8 (α, β, and γ)	6H04 (EM, 5.6 Å)	2,640	–	16
C8 (α, β, and γ)	3OJY (X-ray, 2.51 Å), 6H04 (EM, 5.6 Å)	1,031	10	–
C8 (α, β, and γ)–C9	3OJY-6DLW (EM, 3.9 Å)	2,040	–	5
C9–C9	6H04 (EM, 5.6 Å)	561	3	4

*The individual components analyzed, the reference PDB (method, resolution), number of intra-XLs (threshold < 32 Å), and number of inter-XLs (threshold < 32 Å) are shown in the table columns, respectively. For C9–C9 interaction, detected XLs that could not be explained on a single C9 protein (above threshold), but in C9–C9, were considered as inter-XLs.*

This structural modeling step was done without considering any of the native structures to guide the modeling (blind modeling). Hence, we were able to calculate the Root Mean Square Deviation (RMSD) of the produced model as an average of all backbone atoms (CA, C, N, O) of all pairwise interfaces to the native conformation (reference structures PDB: 6H04 (Menny et al., 2018) at 5.6 Å resolution and PDB: 3OJY (Lovelace et al., 2011) at 2.51 Å resolution). Supplementary Figure 2 shows the detailed RMSD calculations on the pairwise interfaces where the average RMSD over the whole C5b–C9 complex was calculated to be 11.6 Å away from the native conformation. While most of the interfaces were predicted with only a minor deviation from the native conformation, two interfaces (C5b–C6 and C9–C9) increased the average RMSD value. Considering the fact that the MAC is a flexible immune pore complex, this deviation might be either due to conformational differences in the cryoEM maps obtained from MAC complexes assembled from purified components *in vitro*, and the MAC enriched from pooled human plasma, or due to the lower number of detected XLs for these two specific interactions (Table 1).

**FIGURE 3:**
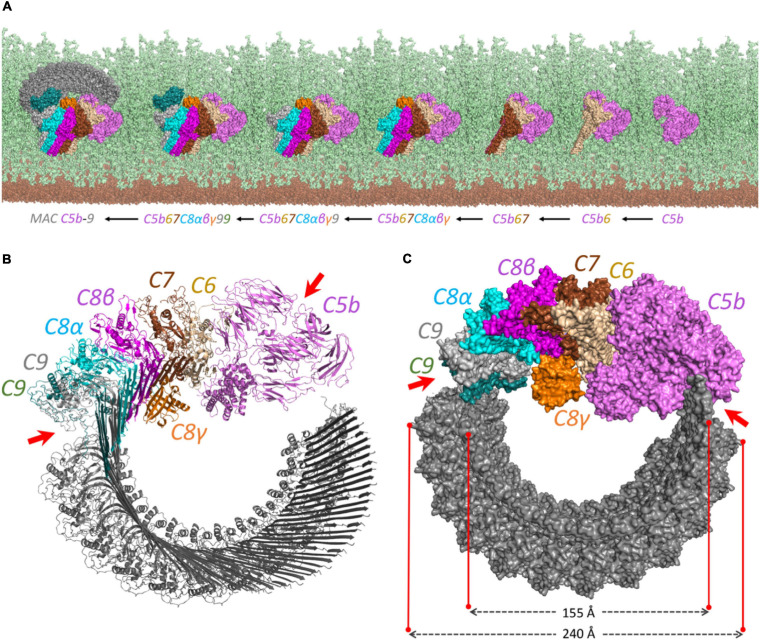
The proposed structural model for the MAC complex. **(A)** The MAC C5b–9 complex formation on the surface of Gram-positive bacteria. It represents how the complement proteins are added one by one and interact together to constitute the MAC complex. The view is from above to show the complete pore formation on the top of the peptidoglycan surface. The cell membrane and peptidoglycan layer are shown in brown and light green, respectively. **(B)** The cartoon view of each component shows how the interaction between C5b and C8 initiates the multiple binding of C9 to form the pore. **(C)** The surface representation. The diagonal length of the inside and the outside of the pore is around 15.5 and 24 nm, respectively. The irregular patterns in C5b–C6 and C9–C9 interactions are shown by red arrows on both **(B)** and **(C)** panels.

The produced pairwise models were further aligned and docked together to constitute the C5b–C9 complex, as indicated in Figure 2B. The predicted complex is supported by a total number of 126 intra- and inter-XLs, as listed in detail in Table 1. While the initial position of C6–C9 represents the formation of C5b–C9 pore, we further employed multiple copies of C9 protein to complete the predicted model of the MAC complex, as represented in Figure 3.

## Discussion

Protein–protein interactions play crucial roles in biological systems yet remained challenging to study. XL-MS, coupled to macromolecular modeling, is a method capable of elucidating high-accuracy models of protein–protein interactions. We previously showed the power of TX-MS method, which is a combinatorial approach based on different MS acquisition techniques and macromolecular modeling to study host–pathogen interactions (Hauri et al., 2019). Here, by representing a simplified and faster version of TX-MS, we applied this method to the many interfaces of the MAC complex while all the complement proteins detected to be highly abundant and associated to the bacterial surface based on DIA-MS analysis. We obtained an estimated average of eight cross-links per interface for the MAC complex, which allowed us to assemble a full model of MAC and compare it with available X-ray crystallography and electron cryo-microscopy models (Lovelace et al., 2011; Serna et al., 2016; Menny et al., 2018).

Here, we elucidate how *in vivo* cross-linking of human plasma to the surface of live bacteria can provide detailed information of protein–protein complexes in their native environment, with possible implications for the studies of conformational differences between *in vitro* crystallized and electron cryo-microscopically imaged and reconstructed macromolecular complexes and *in vivo* cross-linked ones. The approach detailed here should also open up new avenues for the future integration of XL-MS data with *in vivo* prokaryotic electron cryo-tomographic imaging and model reconstruction of host–pathogen complexes (Nans et al., 2014, 2015; Chowdhury et al., 2020).

## Data Availability Statement

The authors confirm that all mass spectrometry mzML files underlying the findings in this study are available from the Zenodo.org database (accession doi: 10.5281/zenodo.4289947).

## Author Contributions

HK, LH, and LM conceived the study. HK and LH wrote the manuscript. HK generated the computational models, analyzed and interpreted the MS data, and wrote the first draft. LH designed and performed the XL-MS sample preparation and analysis. LM, JM, and GT interpreted the data, contributed to the manuscript preparation, and approved the final draft. All authors have read and approved the final manuscript.

## Conflict of Interest

The authors declare that the research was conducted in the absence of any commercial or financial relationships that could be construed as a potential conflict of interest.

## Supplementary Material

Supplementary Figure 1 Detected intra- and inter-XLs were mapped on the experimentally solved structures (PDB ids: 6H04, 3OJY, 4A5W, and 2WII). The two structures above (C3b and C5b) shows how the intra XLs are mapped while structures below mainly represent inter-XLs mapping.

Supplementary Figure 2 RMSD calculation between pairwise docking models and the native structures derived from protein data bank. The average RMSD for each pairwise interaction is calculated between all backbone atoms (CA, C, N, O) and is an average of five PyMOL alignment cycles. The average RMSD for C5b-C6, C6-C7, C7-C8, C8α-C8β, C8(α, β)-C9, and C9-C9 interfaces were 16.06, 6.63, 2.16, 0.97, 15.19, and 28.76 Å, respectively. The outliers here are C5b-C6, and C9-C9 interfaces where we see a rotation on C5b and a shift in C9 resulting in high RMSD values (indicated with red arrows).
