# Supplementary figures and images for: In vivo Cross-Linking MS of the Complement System MAC Assembled on Live Gram-Positive Bacteria
Source: Front Genet. 2021 Jan 8;11:612475. doi: 10.3389/fgene.2020.612475 (PMC7820895; doi:10.3389/fgene.2020.612475)

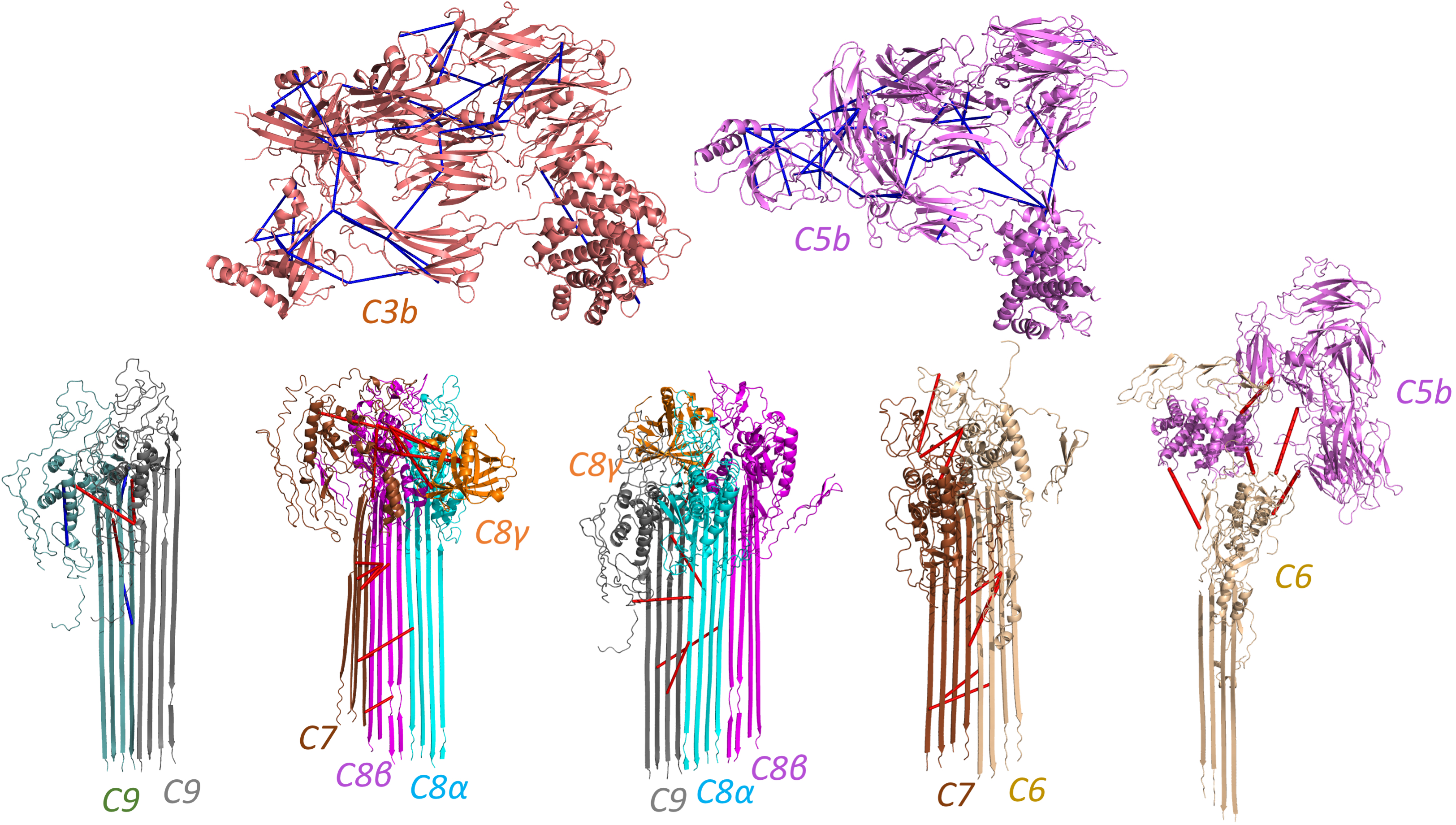

Supplement: Supplementary Figure 1 — Detected intra- and inter-XLs were mapped on the experimentally solved structures (PDB ids: 6H04, 3OJY, 4A5W, and 2WII). The two structures above (C3b and C5b) shows how the intra XLs are mapped while structures below mainly represent inter-XLs mapping. [file Figure_1.JPEG]

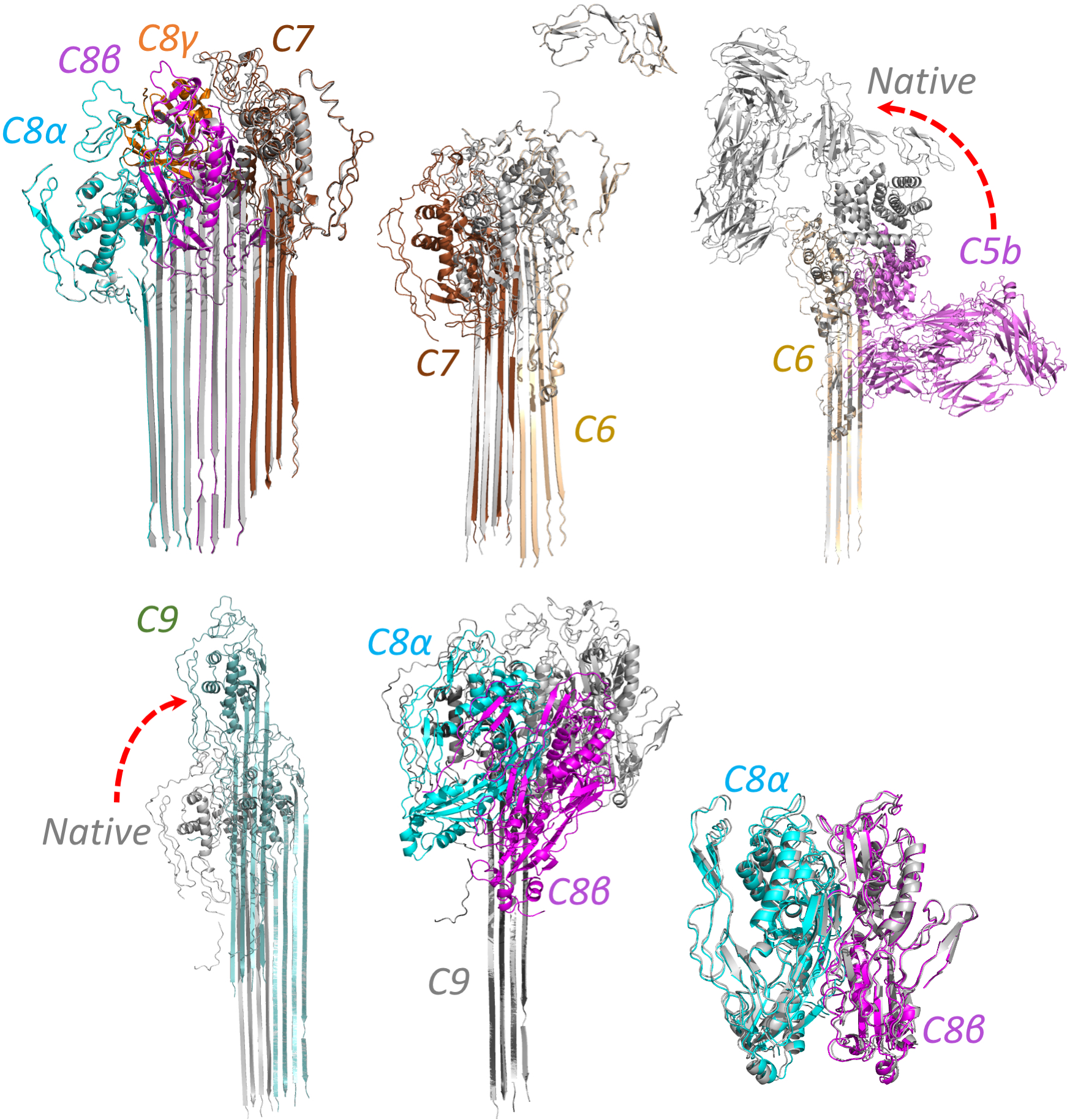

Supplement: Supplementary Figure 2 — RMSD calculation between pairwise docking models and the native structures derived from protein data bank. The average RMSD for each pairwise interaction is calculated between all backbone atoms (CA, C, N, O) and is an average of five PyMOL alignment cycles. The average RMSD for C5b-C6, C6-C7, C7-C8, C8α-C8β, C8(α, β)-C9, and C9-C9 interfaces were 16.06, 6.63, 2.16, 0.97, 15.19, and 28.76 Å, respectively. The outliers here are C5b-C6, and C9-C9 interfaces where we see a rotation on C5b and a shift in C9 resulting in high RMSD values (indicated with red arrows). [file Figure_2.JPEG]
